# Brachial plexopathy and intradural cord compression caused by malignant peripheral nerve sheath tumor a case report and literature review

**DOI:** 10.1016/j.ijscr.2024.110610

**Published:** 2024-11-14

**Authors:** Ahmad Alelaumi, Almu'Atasim Khamees, Mohammad Alfawareh, Osama Khalil, Anas Zahran

**Affiliations:** a Department of Orthopedics and Spine Surgery, King Hussein Cancer Center, Amman, Jordan; b Department of Surgery, King Hussein Cancer Center, Amman, Jordan; c Department of Rehabilitation, King Hussein Cancer Center, Amman, Jordan

**Keywords:** Case report, Brachial plexopathy, Cancer, Radiation

## Abstract

**Introduction and importance:**

Diagnosing brachial plexopathy in cancer patients who have undergone treatment and are being monitored presents a significant difficulty due to the potential involvement of multiple factors, including tumor recurrence causing compression or infiltration, recurrent metastasis, or the effects of radiation therapy. Malignant peripheral nerve sheath tumors (MPNSTs) have the potential to impact the brachial plexus, resulting in brachial plexopathy. Misdiagnosis can lead to catastrophic outcomes.

**Case presentation:**

A 29-year-old female patient, who had a previous history of nasopharyngeal carcinoma, exhibited symptoms consistent with brachial plexopathy. The primary diagnoses for the cause were tumor metastatic recurrence and radiation-induced brachial plexopathy. Following an evaluation, recurrence appeared to be the most probable diagnosis. The mass had infiltrated along the brachial plexus, resulting in an intradural mass that led to cord compression. The final pathology report confirmed that the original pathology was malignant peripheral nerve sheath tumor (MPNST).

**Clinical discussion:**

Understanding the underlying causes of brachial plexopathy is crucial for accurate diagnosis, particularly in cancer patients and those with a history of radiotherapy, as these individuals may present with complex or atypical symptoms that can complicate the diagnostic process. In such cases, distinguishing between tumor-related brachial plexopathy, radiation-induced nerve damage, and other potential etiologies is essential for guiding appropriate treatment strategies and improving patient outcomes.

**Conclusions:**

Comprehensive and prompt evaluation is crucial in cases of brachial plexopathy with a history of cancer, aiming to prevent misdiagnosis and minimize complications.

## Highlights

• The primary factors contributing to brachial plexopathy in individuals with cancer are typically radiation-induced damage or tumor compression/infiltration. It is crucial to consider the possibility of primary tumors affecting the brachial plexus, particularly when there is a remote history of cancer or radiation therapy.
• Radiation treatment can occasionally lead to the development of malignant peripheral nerve sheath tumors (MPNSTs), although it is uncommon for them to affect the brachial plexus. Furthermore, it is rare for these tumors to extend along the brachial plexus to the extent of causing cord compression.
• This study emphasizes the importance of conducting a thorough evaluation in cases of brachial plexopathy among cancer patients. Whenever feasible, it is crucial to consider various potential causes for the condition.
• Attaining a diagnosis should involve various modalities in cases of brachial plexopathy.
• In cases of brachial plexopathy in cancer patients, it is advisable to always consider performing a biopsy. This approach has the potential to prevent catastrophic outcomes and ensure the best possible outcome for the patient.

## 1 Introduction

The upper limb receives motor and sensory innervation from the brachial plexus. Brachial plexopathy (BP) is a type of peripheral neuropathy that specifically impacts the brachial plexus. Among individuals with cancer, the occurrence of brachial plexopathy is estimated to be around 0.4 %, recurrent metastatic disease is a significant cause [1]. Brachial plexopathy is more commonly observed in cancer patients who have undergone radiation treatment (radiation induced brachial plexopathy), and its prevalence can differ depending on factors such as tumor type, the specific radiotherapy method used, and the tumor's location. According to a study by Rudra et al., the occurrence of BP in breast cancer patients who received radiation therapy ranged from 0.4 % to 1.6 %. It has been noted that newer radiotherapy techniques like inverse planning intensity modulated radiation therapy (IMRT) have been associated with lower rates of BP [2]. In a study conducted by Olsen et al. in 1993, it was reported that the incidence of radiation induced brachial plexopathy (RIBP) was higher, ranging from 5 % to 9 %. This higher occurrence could be attributed to various factors such as the administration of cytotoxic chemotherapy and the utilization of older techniques of radiotherapy [3]. In the case of radiation-treated nasopharyngeal carcinomas (NPC), the reported incidence of brachial plexopathy was found to be approximately 0.3 %. This suggests that, in general, the occurrence of brachial plexopathy in this particular context is lower compared to other instances of radiation-induced brachial plexopathy [4]. A retrospective review conducted by Cai et al. examined 31 patients with brachial plexopathy resulting from radiation-treated nasopharyngeal carcinoma (NPC) cases. he primary symptom observed among these patients was paresthesia. Magnetic resonance imaging (MRI) revealed hyper-intensity on T1, T2, and post-contrast T1 images [5]. Malignant peripheral nerve sheath tumors (MPNSTs) are aggressive, high-grade malignant spindle-cell neoplasms. These tumors often exhibit a variable Schwann cell phenotype and have the potential to arise from pre-existing plexiform neurofibromas [6]. MPNSTs can occur in individuals with Neurofibromatosis type 1 (NF1), with a lifetime risk of approximately 10 % for developing this type of tumor. Approximately 45 % of MPNST cases manifest spontaneously, meaning they arise without a known cause. However, in some cases, MPNSTs can emerge as a result of radiation exposure. The incidence of radiation-induced MPNSTs in large series ranges from 5.5 % to 11 % [7]. MPNSTs have the potential to affect the brachial plexus, and due to their tendency to invade the surrounding soft tissues, they can present as a brachial plexopathy. However, in cases where patients have received radiation treatment, there can be a significant challenge in distinguishing between radiation-induced brachial plexopathy and MPNST of the brachial plexus.

MPNST of the brachial plexus is a rare occurrence, but multiple case reports can be found in the literature documenting such cases. For instance, Donaldson et al. reported three cases of MPNST involving the brachial plexus over a period of six years. Similarly, Pressney et al. reported thirteen cases of MPNST of the brachial plexus over a span of 12.5 years. These cases highlight the existence of MPNST as a potential cause of brachial plexopathy [8,9].

.Delay or misdiagnosis of MPNST involving the brachial plexus can have severe consequences, as illustrated in the presented case. In our case, growth, and infiltration of the MPNST along the brachial plexus roots, progressed proximally to reach the cord, resulted in cord compression and subsequent lower limb weakness due to the presence of an intradural extramedullary mass.

In situations where patients present with brachial plexopathy and have a history of radiation treatment, it becomes crucial to achieve an early and accurate diagnosis to prevent catastrophic outcomes. Obtaining a tissue diagnosis through appropriate diagnostic procedures should always be considered. And to highlight the importance of that we present our case which was managed in tertiary academic cancer center in Jordan.

## 2 Case description

A 29-year-old female patient with a previous medical history of hypothyroidism came in with a gradual worsening of weakness over a period of 6 months, specifically affecting her entire right upper limb. She experienced neck pain, and intermittent needle-like pain in her right upper limb, which she rated as a 3–4 on the visual analog scale (VAS), and the pain was most prominent during nighttime. The pain primarily occurred in the distribution of the C5 and C6 dermatomes, and to a lesser extent, the C7 dermatome. At the time of her visit, the aforementioned symptoms had become significantly worse. The VAS score increased to 6–7 points, and the frequency of the pain had also intensified. Consequently, she was unable to sleep. The patient had a history of nasopharyngeal carcinoma (NPC) and had undergone standard radiotherapy and chemotherapy a decade prior to her current presentation.

Clinical examination showed reduced muscle strength and muscle wasting in her left upper limb. Deltoid muscle power was 0/5, biceps muscle was 3/5, wrist extension was 3/5, triceps muscle and hand intrinsic muscles were 4/5. There was hyposthesia in C5 and C6 dermatomal distribution. The right biceps and radial reflexes did not elicit a response, but the rest of the neurological examination appeared normal.

Differential diagnosis included viral induced brachial plexopathy and tumor metastatic recurrence. Electrophysiological studies were conducted to assess the brachial plexus. Nerve conduction studies revealed that the motor and sensory nerves of the left brachial plexus showed signs of injury, primarily axonal damage. Furthermore, needle electromyography indicated damage to the left brachial plexus.

The patient underwent a whole-body positron emission tomography PET CT two months prior to the spine clinic presentation due to her neck pain. The PET CT scan revealed a newly identified area with abnormal increased fluorodeoxyglucose (FDG) uptake, reaching a maximum SUV value of 7.05. This finding can be linked to the CT results, which showed thickening of the soft tissue surrounding the right side of the C5 vertebra. Additionally, the cervical spine MRI indicated thickening and enhancement of the right C4–5 nerve roots.

**Fig. 1:**
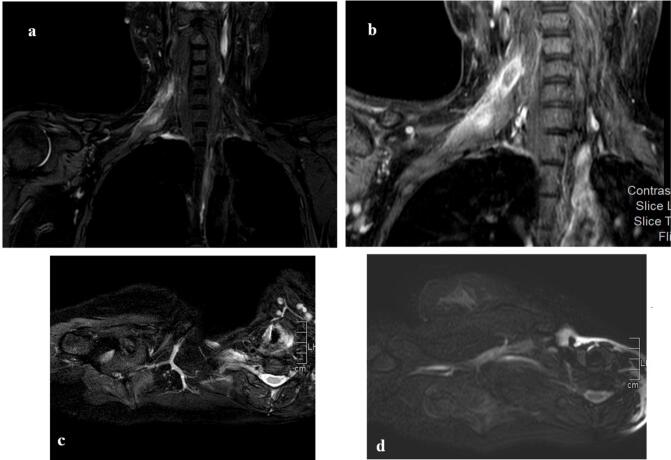
Fig. (1-a) T2 weighted brachial plexus MRI coronal cut. Fig. (1-b) T2 weighted brachial plexus MRI coronal cut. Fig. (1-c) T2 weighted brachial plexus MRI axial cut. Fig. (1-d) T2-weighted short-tau inversion recovery (STIR) brachial plexus MRI axial cut. Showing smooth thickening, hyperintense signal on fluid sensitive images and diffuse postcontrast enhancement involving C5 and to lesser extent C6 spinal roots, extending to the upper trunks, divisions, lateral and posterior cords of the right brachial plexus (red arrows). (For interpretation of the references to colour in this figure legend, the reader is referred to the web version of this article.)

Following the clinic presentation, a comprehensive evaluation was conducted, including laboratory tests which were normal. Subsequently, further neuroimaging was undertaken. The brachial plexus was assessed using magnetic resonance imaging (MRI), revealing a uniform thickening and enhancement affecting the C5 and, to a lesser extent, C6 nerve roots. This condition extended to the upper trunks, divisions, lateral and posterior cords of the right brachial plexus. The report concluded that these observations may be indicative of neuritis associated with radiation treatment, emphasizing the need for clinical correlation and subsequent follow-up scans Fig. 1.

Considering the patient's gradual and progressive symptom onset, starting with pain in the right upper limb and subsequently leading to progressive weakness affecting the entire upper limb function, and taking into account her past history of tumor treatment, the potential diagnoses being considered were radiation-induced brachial plexopathy (RIBP) or recurrent metastasis of nasopharyngeal carcinoma (NPC).

The tumor board convened to discuss the case, taking into consideration the patient's 10-year history of disease-free follow-ups and the absence of any significant findings in the latest PET-CT scan, except for the previously mentioned increased uptake. Based on these factors, the most probable explanation was determined to be late-onset radiation-induced brachial plexopathy (RIBP).

The patient continued to receive regular care in the neurology clinic, attending multiple appointments. A trial of steroid treatment was initiated but discontinued since the patient did not experience any noticeable improvement in her symptoms. After a follow-up period of three months, the neurology team concluded that the most probable explanation for the condition was metastatic recurrence in the brachial plexus. Consequently, the patient was referred back to the oncology team for further management.

**Fig. 2:**
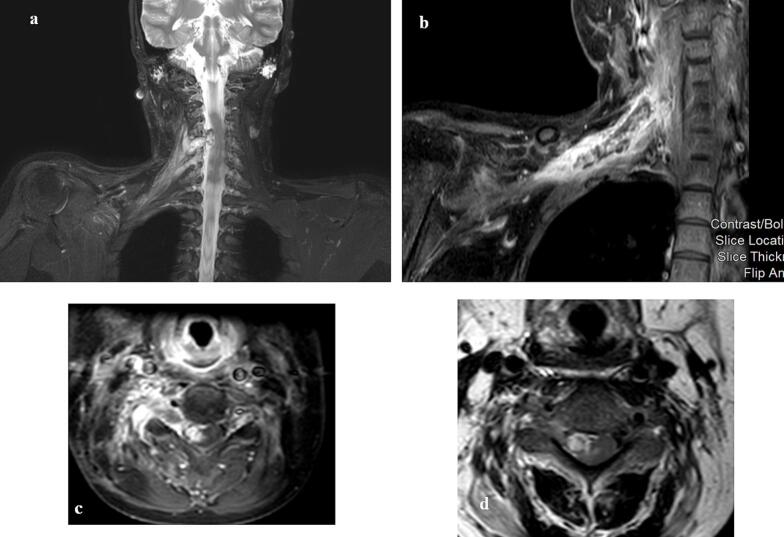
Fig. (2-a) T2 weighted brachial plexus MRI coronal cut. Fig. (2-b) T1 weighted STIR brachial plexus MRI coronal cut. Fig. (2-c) T1 weighted STIR cervical spine MRI axial cut. Fig. (2-d) T2 weighted cervical spine MRI axial cut showing progression of the thickening with lobulated appearances involving the nerve roots at C4-C5 and C5-C6 levels on the right side with brachial plexus involvement and intraspinal extension medially best appreciated at the level of the C4-C5 neural foramina (red arrow) resulting in mass effect upon at the corresponding portion of the spinal cord and cord displacement to the left side. (For interpretation of the references to colour in this figure legend, the reader is referred to the web version of this article.)

The oncology team requested new neuroimaging studies, including neck MRI, cervical spine MRI, and brachial plexus MRI. The results revealed no signs of nasopharyngeal tumor recurrence or newly enlarged lymph nodes. However, there was observed interval progression of neural thickening with lobulated appearances, primarily affecting the C4-C5 and C5-C6 exiting nerve roots on the right side. The involvement of the brachial plexus extended laterally up to the lateral third of the right clavicle, as well as medially with intraspinal extension, most prominent at the level of the C4-C5 neural foramina on the right side. These findings resulted in mass effect on the corresponding segment of the spinal cord and displacement of the cord towards the left side. As a result, these concluded as a strong indication for the presence of a metastatic process involving the nerves Fig. 2.

A repeat PET-CT scan was conducted, which indicated a notable advancement of a hypermetabolic lesion within the neural foramina of the C4 and C5 vertebrae. The lesion extended through the right nerve roots and into the brachial plexus, aligning with the recent findings from the brachial plexus MRI.

The patient's clinical condition deteriorated significantly, experiencing severe and uncontrollable pain, along with progressive weakness in all muscle groups of the right upper limbs, resulting in a power of 0/5. Due to the imminent risk of paralysis caused by cord compression, the patient was admitted and initiated on dexamethasone treatment.

Upon the patient's admission, she experienced a sudden onset of bilateral lower limb weakness. Due to the urgency of the situation, she was promptly admitted to the operation room for surgical decompression. During this procedure, a laminectomy was performed at C5 and C6 levels and a gross total resection of the intradural mass was achieved. The excised mass was then sent for pathology examination to determine its nature and provide a definitive diagnosis.

The following day after the surgery, there was a significant improvement in the patient's lower limb weakness. She regained the ability to walk and her condition continued to gradually improve. Within one week, she progressed to the point where she could walk without the need for any support or assistance.

The histopathologic examination of the excised tumor revealed a diagnosis of MPNST. The tumor exhibited alternating hypocellular and hypercellular areas composed of uniform spindle cells. These spindle cells displayed hyperchromatic thin wavy nuclei, prominent mitotic activity, and areas of necrosis. The stroma of the tumor showed a myxoid appearance.

Immunohistochemical analysis revealed that the tumor cells were positive for CD56, which is a marker for neural differentiation. However, the tumor cells were negative for PanCytokeratin, CK5/6, GFAP, SS18, S100, SOX10, SMA, Desmin, CD34, STAT6, and Synaptophysin. These markers were tested to exclude other possible tumor types and to support the diagnosis of MPNST.

**Fig. 3:**
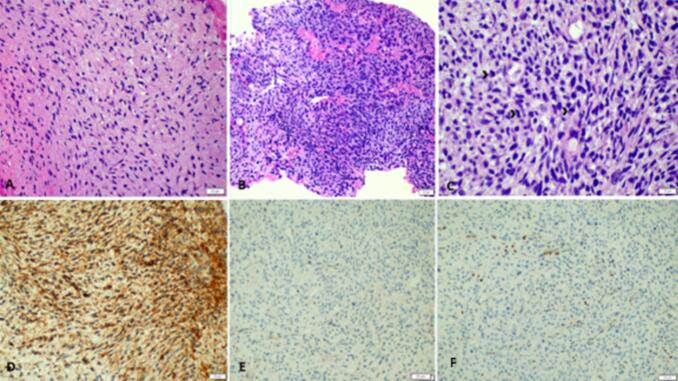
Histopathologic examination revealed alternating hypocellular (A) and hypercellular areas (B) (H&E 10×), (C) spindle cells with hyperchromatic thin wavy nuclei, prominent mitotic activity (arrow head) (H&E 40×). (D) The tumor cells are positive for CD56. (E) S100 is negative. (F) H3K27me3 immunostain shows loss of nuclear staining in the tumor cells in the presence of positive internal control.

A characteristic immunostain for H3K27me3 was performed, showing a loss of nuclear staining in the tumor cells while demonstrating positive staining in the internal control of endothelial cells. This immunostain has good sensitivity and robust specificity for the diagnosis of MPNST, further supporting the diagnosis in this case [10] Fig. 3.

**Fig. 4:**
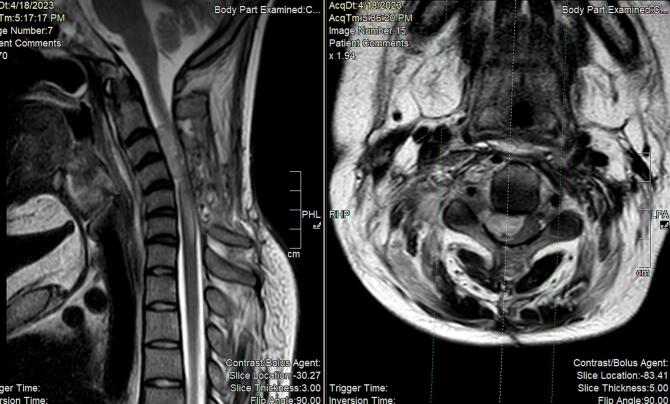
Fig. (4-a) T2 weighted cervical spine MRI sagittal cut showing. Fig. (4-b) T2 weighted cervical spine MRI axial cut showing mass extension along C3-C4 right exit foramen and a new epidural mass measuring 2.9 × 1.5 cm in length along C3 and C4 levels. The mass is causing compression upon the spinal cord at this level.

Following the pathology results, the case was discussed in the tumor board, and the decision was made to initiate chemotherapy as part of the treatment plan. However, before starting chemotherapy, the patient presented again, one month after the surgery, with the same complaint of bilateral lower limb weakness. A cervical spine MRI was performed, revealing a recurrence of the lesion at the C3 and C4 levels, causing compression of the spinal cord Fig. 4.

In an attempt to control the disease, a trial of palliative radiotherapy was conducted, but unfortunately, it was not successful. The patient's condition further deteriorated, and she experienced an episode of aspiration, which resulted in aspiration pneumonia. Despite medical interventions, her condition continued to decline, and tragically, she passed away after a few days.

It is a sad outcome that highlights the aggressive nature of the disease and the challenges associated with managing MPNST with spinal cord compression. Despite the efforts made in treatment, the progression of the disease and its complications ultimately led to a tragic outcome.

## 3 Discussion

Brachial plexopathy is a debilitating condition that can result in pain, weakness, numbness, and loss of motor control in the affected limb. Understanding the underlying causes of brachial plexopathy is crucial for accurate diagnosis, effective treatment, and prevention. Some common causes of brachial plexopathy include traumatic injury, repetitive motion, tumors, radiation therapy, and inflammatory/autoimmune disorders.

Traumatic injuries, such as motor vehicle accidents, falls, or sports-related incidents, are a leading cause of brachial plexopathy. These injuries can cause direct trauma to the brachial plexus, leading to nerve compression, stretching, or tearing. Repetitive motion or overuse injuries can also contribute to the development of brachial plexopathy, particularly in occupations or activities involving repetitive arm and shoulder movements [11].

Tumors or abnormal growths in the vicinity of the brachial plexus can compress or infiltrate the nerves, causing brachial plexopathy. Both benign and malignant tumors can lead to nerve damage. Radiation therapy can cause inflammation, scarring, nerve damage, leading to radiation-induced brachial plexopathy.

Inflammatory and autoimmune disorders, such as brachial neuritis (Parsonage-Turner syndrome), can also lead to brachial plexopathy. The exact cause of brachial neuritis is not fully understood, but it involves an immune-mediated response that triggers nerve inflammation and damage [12].

Brachial plexopathy caused by tumors is commonly due to tumor infiltration or compression of the nerves. Various types of cancer can potentially cause brachial plexopathy, including lung cancer, breast cancer, lymphoma, and sarcomas. Additionally, radiation therapy and surgical procedures in the vicinity of the brachial plexus can also contribute to nerve damage.

The symptoms of brachial plexopathy vary depending on the location and severity of nerve involvement. Patients may experience pain, weakness, numbness, tingling, difficulty with fine motor skills and coordination, and muscle atrophy in severe cases.

Diagnosing brachial plexopathy in cancer patients involves a thorough clinical evaluation, including a detailed medical history, physical examination, electrodiagnostic studies, and imaging studies. Magnetic resonance imaging (MRI) is often employed to visualize the brachial plexus and identify any abnormalities or tumor involvement.

Management of brachial plexopathy in cancer patients is typically focused on treating the underlying cause while providing symptomatic relief. Treatment approaches depend on the extent of nerve damage and the underlying cancer and may involve chemotherapy, radiation therapy, surgical intervention, or a combination of these modalities.

Radiation-induced brachial plexopathy (RIBP) can also occur in cancer patients who underwent radiotherapy, it is a chronic condition that develops gradually, typically months to years after radiation treatment. The exact pathophysiology of RIBP is not fully understood but involves damage to blood vessels supplying the brachial plexus, leading to reduced blood flow and subsequent nerve injury. Radiation can also directly damage nerve tissue, causing inflammation, fibrosis, and impaired nerve function [13].

The management of RIBP focuses on alleviating pain and improving function through a multidisciplinary approach, including pain management strategies, physical therapy, occupational therapy, and assistive devices to support daily activities. Medications such as analgesics, neuropathic pain medications, and anti-inflammatory drugs may be prescribed to manage pain and reduce inflammation.

In the case we presented, the management approach involved considering both RIBP and recurrent metastasis as differential diagnoses until the pathology results revealed the presence of MPNST. This highlights the importance of performing a biopsy to obtain a definitive diagnosis. It allows for a more accurate understanding of the underlying pathology and helps to tailor the management plan accordingly. This study encourages the formulation of treatment algorithms for brachial plexopathy in cancer patients.

Finally, we would like to state that this work has been reported in line with the SCARE criteria [14].

## Informed consent

Written informed consent was obtained from the patient for publication of medical information and accompanying images. A copy of the written consent is available for review by the Editor-in-Chief of this journal on request.

## Ethical approval

Ethical approval was waived by the institutional review board (IRB) committee in King Hussein Cancer Center, Amman, Jordan.

## Guarantor

Ahmad Alelaumi.

## Research registration number

The study is showing a unique presentation with diagnostic dilemma but not “First in Man”.

## Funding

The authors received no financial support for the research, authorship, and/or publication of this article.

## Author contribution

The authors confirm contribution to the paper as follows: data collection: all authors; analysis and; draft manuscript preparation: All authors. All authors reviewed the results and approved the final version of the manuscript.

## Conflict of interest statement

The authors declared no potential conflicts of interest with respect to the research, authorship, and/or publication of this article.

## Data Availability

The data that supports the findings of this study are available in the supporting information of this article.

## References

[1] Jaeckle K.A. (Dec 2004). Neurological manifestations of neoplastic and radiation-induced plexopathies. Semin. Neurol..

[2] Rudra S., Roy A., Brenneman R., Gabani P., Roach M.C., Ochoa L., Prather H., Appleton C., Margenthaler J., Peterson L.L., Bagegni N.A., Zoberi J.E., Garcia-Ramirez J., Thomas M.A., Zoberi I. (Oct 27 2020). Radiation-induced brachial plexopathy in patients with breast cancer treated with comprehensive adjuvant radiation therapy. Adv. Radiat. Oncol..

[3] Olsen N.K., Pfeiffer P., Johannsen L., Schrøder H., Rose C. (Apr 30 1993). Radiation-induced brachial plexopathy: neurological follow-up in 161 recurrence-free breast cancer patients. Int. J. Radiat. Oncol. Biol. Phys..

[4] Tuan J.K., Ha T.C., Ong W.S., Siow T.R., Tham I.W., Yap S.P., Tan T.W., Chua E.T., Fong K.W., Wee J.T. (Sep 2012). Late toxicities after conventional radiation therapy alone for nasopharyngeal carcinoma. Radiother. Oncol..

[5] Cai Z., Li Y., Hu Z., Fu R., Rong X., Wu R., Cheng J., Huang X., Luo J., Tang Y. (Apr 5 2016). Radiation-induced brachial plexopathy in patients with nasopharyngeal carcinoma: a retrospective study. Oncotarget.

[6] Belakhoua S.M., Rodriguez F.J. (Feb 16 2021). Diagnostic pathology of tumors of peripheral nerve. Neurosurgery.

[7] Gupta G., Mammis A., Maniker A. (Oct 2008). Malignant peripheral nerve sheath tumors. Neurosurg. Clin. N. Am..

[8] Donaldson E.K., Winter J.M., Chandler R.M., Clark T.A., Giuffre J.L. (Apr 1 2023). Malignant peripheral nerve sheath tumors of the brachial plexus: a single-center experience on diagnosis, management, and outcomes. Ann. Plast. Surg..

[9] Pressney I., Khoo M., Khan R., Abernethy P., Hargunani R., Saifuddin A. (Aug 2021). Morphology of the entering and exiting nerve as a differentiating feature of benign from malignant peripheral nerve sheath tumours of the brachial plexus. Skeletal Radiol..

[10] Prieto-Granada C.N., Wiesner T., Messina J.L., Jungbluth A.A., Chi P., Antonescu C.R. (Apr 2016). Loss of H3K27me3 expression is a highly sensitive marker for sporadic and radiation-induced MPNST. Am. J. Surg. Pathol..

[11] Dumitru D., Amato A.A., Zwarts M.J. (2002). Electrodiagnostic Medicine.

[12] Sumner A.J. (Oct 2009). Idiopathic brachial neuritis. Neurosurgery.

[13] Delanian S., Lefaix J.L., Pradat P.F. (Dec 2012). Radiation-induced neuropathy in cancer survivors. Radiother. Oncol..

[14] Sohrabi C., Mathew G., Maria N., Kerwan A., Franchi T., Agha R.A. (2023). The SCARE 2023 guideline: updating consensus surgical CAse REport (SCARE) guidelines. Int. J. Surg. Lond. Engl..

